# Association between magnesium depletion score and prevalence and all-cause mortality of psoriasis among the US population

**DOI:** 10.3389/fnut.2025.1598688

**Published:** 2025-09-11

**Authors:** Lin Qi, Xian Yang

**Affiliations:** The First Hospital of Hunan University of Chinese Medicine, Changsha, China

**Keywords:** magnesium, magnesium depletion score, psoriasis, cross-sectional study, NHANES

## Abstract

**Background:**

Magnesium plays a key role in the physiopathologic process of psoriasis. The recently proposed magnesium depletion score (MDS) represents a comprehensive index for assessing magnesium status. However, the effect of MDS on psoriasis remains to be elucidated. This study aimed to evaluate the possible association between MDS and psoriasis prevalence and mortality.

**Methods:**

This study utilized data from the National Health and Nutrition Examination Survey (NHANES) of adult participants. The multivariable logistic regression analysis was employed to assess the relationship between MDS and psoriasis prevalence. Restricted cubic splines (RCS) were utilized to investigate the dose–response correlation. Furthermore, Cox regression analysis was performed to determine the relationship between MDS and all-cause mortality in psoriasis patients. Furthermore, we conducted subgroup and sensitivity analyses to verify the validity and consistency of these results.

**Results:**

This study enrolled 17,883 eligible participants. After excluding individuals without follow-up information, 64 all-cause deaths were observed among 505 patients with psoriasis. In the weighted multivariable logistic regression model, individuals with an MDS ≥ 3 had a 1.75-fold greater risk of psoriasis compared with those with an MDS of 0 (OR = 1.75; 95% CI: 1.05–2.92; *p* < 0.05). RCS analysis revealed a positive linear relationship between MDS and psoriasis prevalence (*P* for nonlinear = 0.145). Additionally, Cox regression analysis demonstrated that MDS was positively associated with all-cause mortality (HR = 1.39; 95% CI: 1.04–1.87; *p* < 0.05). Subgroup analyses indicated that these findings remained consistent across different subgroups.

**Conclusion:**

MDS is associated with an increased prevalence and all-cause mortality from psoriasis among American adults. Early detection and management for MDS may reduce the risk of psoriasis and improving its prognosis.

## 1 Introduction

Psoriasis is an immune-mediated chronic inflammatory skin disorder with a global prevalence of approximately 2–3% (1, 2). It is distinguished by erythematous papules covered with silvery-white scales, commonly affecting the scalp, back, and extensor surfaces of the limbs. Beyond cutaneous manifestations, psoriasis can also impact multiple organ systems. Patients with psoriasis commonly have comorbidities, including psoriatic arthritis (3), diabetes (4), and cardiovascular diseases (5, 6). Psoriasis imposes significant physical and psychological trauma on patients, severely compromising their quality of life (7). It also imposes significant costs on individuals and healthcare systems. However, the pathogenesis of psoriasis remains incompletely understood. Current evidence indicates that its etiology is caused by genetic vulnerability, immune dysregulation, and environmental factors.

Magnesium, a crucial regulatory ion in numerous enzymatic reactions, is essential for human health and disease prevention (8). As the second common intracellular cation in the human body, magnesium ions are required for normal cellular activity and physiological homeostasis (9). Furthermore, magnesium participates in a variety of enzymatic reactions, having a crucial effect on energy metabolism, maintaining immune balance, and modulating oxidative stress and inflammatory responses (10). Magnesium deficiency or dysregulation of magnesium metabolism is implicated in the pathological processes of various diseases (11).

In recent years, the role of micronutrients in psoriasis has garnered increasing attention. Growing evidence suggests that micronutrients such as calcium, magnesium, zinc, and selenium influence the onset and progression of psoriasis through various biological mechanisms (12, 13). Previous studies have mainly examined the effect of serum magnesium levels and dietary magnesium intake on the disease. However, these evaluation criteria frequently ignore aspects such as renal excretion and intracellular storage, thereby failing to sufficiently capture magnesium level. Consequently, the prevalence of magnesium deficiency and its potential health effects may be significantly underestimated. The relationship between magnesium homeostasis and health or disease remains insufficiently explored. There is an urgent need to develop a precise, simple, and widely applicable tool for assessing magnesium bioavailability.

The magnesium depletion score (MDS) represents a newly proposed composite indicator that measures magnesium status in the body (14). MDS comprehensively evaluates four crucial risk factors, including proton pump inhibitor (PPI) and diuretic use, alcohol consumption, and renal disease. MDS takes into account renal reabsorption function, serving as a sensitive and reliable indicator for assessing magnesium deficiency status in the human body. A higher MDS means more severe magnesium insufficiency. MDS has been found to be strongly related to the risk and prognosis of several various diseases (15, 16). Nonetheless, the relationship between MDS and psoriasis remains to be explored. In view of this, this study evaluated the potential association between MDS and psoriasis based on data from the National Health and Nutrition Examination Survey (NHANES). The objective of this study was to reveal the relationship between magnesium homeostasis and psoriasis prevalence and mortality. These findings are expected to expand the understanding of environmental risk factors for psoriasis and open new perspectives for individualized nutritional management.

## 2 Materials and methods

### 2.1 Study population

**Figure 1:**
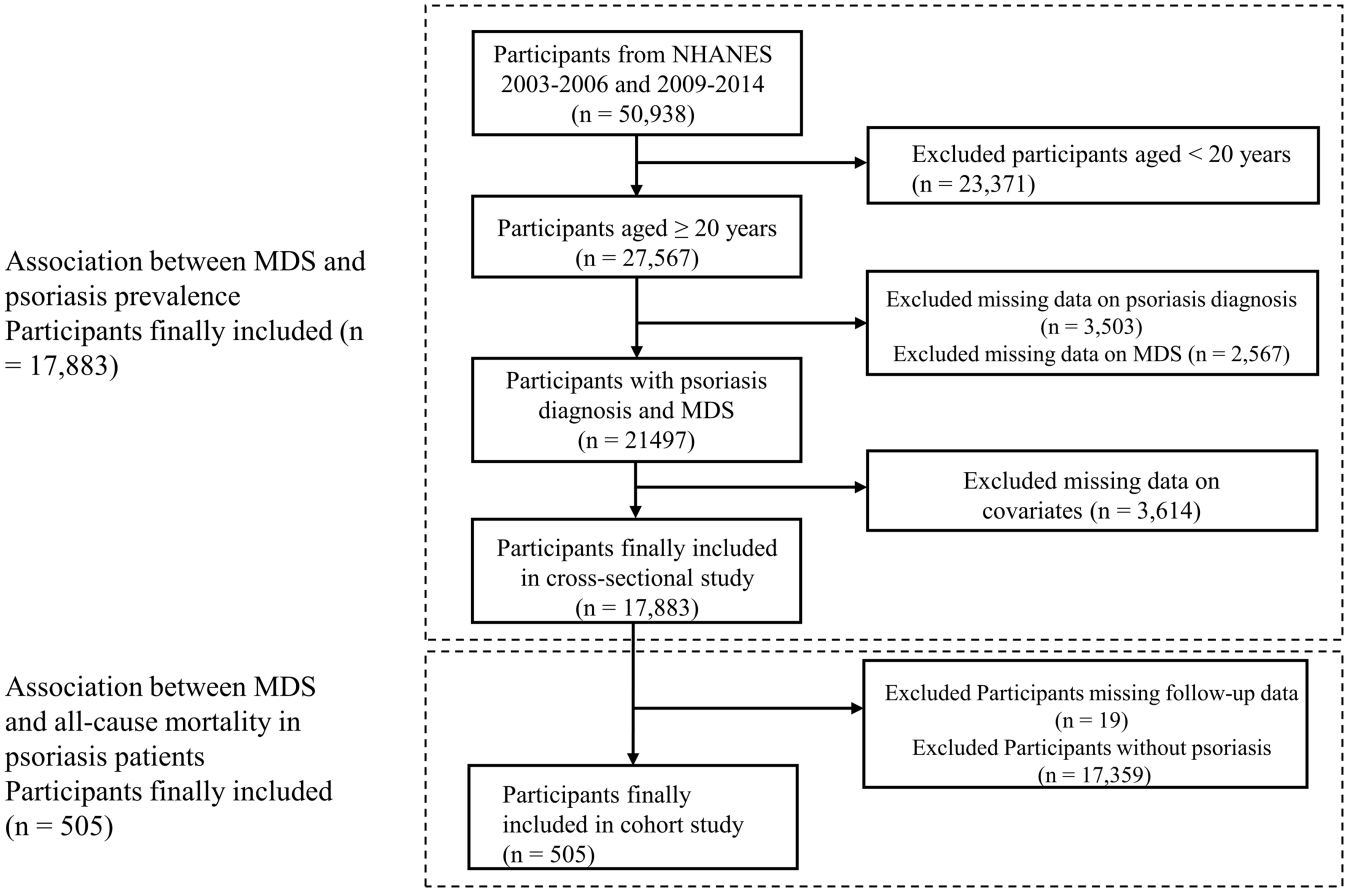
Flowchart for participant selection process. MDS, magnesium depletion score; PIR, poverty income ratio; BMI, body mass index.

Participant data were obtained from NHANES, a publicly accessible database in the United States. The NHANES protocol was approved by the Research Ethics Review Board of the National Center for Health Statistics (NCHS), and written informed consent was obtained from each participant. This study utilized data from five NHANES cycles (2003–2004, 2005–2006, 2009–2010, 2011–2012, and 2013–2014). These cycles were selected because psoriasis data were only recorded in these five cycles within NHANES. The participant screening process is illustrated in Figure 1. The study included adults aged 20 years and above. Initially, 50,938 participants were recruited. Through a rigorous screening process, we excluded individuals under 20 years of age (n = 23,371), participants lacking psoriasis questionnaire information (n = 3,503), and those without MDS information (n = 2,567). Furthermore, we excluded those without information on covariates (n = 3,614). Ultimately, data from 17,883 eligible participants were analyzed to determine the relationship between MDS and psoriasis prevalence. To assess the influence of MDS on mortality among those with psoriasis, we excluded individuals missing follow-up information (n = 19) and non-psoriasis participants (n = 17,359). Finally, data from 505 psoriasis patients were analyzed to identify the connection between MDS and psoriasis mortality.

### 2.2 Assessment of MDS

MDS served as an exposure variable in this study. MDS was calculated following the approach developed by Fan et al. (14). MDS is estimated by a composite score of four major elements. The current use of diuretics or proton pump inhibitors (PPI) is assigned one point. Excessive alcohol intake (defined as >2 drinks/day for males and >1 drink/day for females) is scored one point. An estimated glomerular filtration rate (eGFR) between 60 and 90 mL/min/1.73 m^2^ is scored one point, while an eGFR below 60 mL/min/1.73 m^2^ is scored two points. The eGFR is estimated by the Chronic Kidney Disease Epidemiology Collaboration equation, based on serum creatinine levels (17). A higher MDS indicates a greater risk of magnesium deficiency. Consistent with previous studies (16), in addition to continuous MDS variable, MDS is categorized into four groups as a categorical variable for analysis: 0, 1, 2, and ≥3.

### 2.3 Assessment of psoriasis

Psoriasis was the outcome variable in this study. Psoriasis is diagnosed by professional dermatologists through careful morphological assessment of skin lesions. Trained interviewers asked participants “Have you ever been informed by a healthcare provider that you have psoriasis?” Those who responded “yes” were categorized as suffering from psoriasis. We excluded participants who refused to answer or were unsure.

### 2.4 Covariates

To minimize the impact of confounding variables, we adjusted for potential covariates based on prior literature and clinical knowledge (18). The covariates considered in this study included age, sex, race/ethnicity, education, poverty-to-income ratio (PIR), body mass index (BMI), smoking status, alcohol intake, diabetes, coronary heart disease (CHD), and dietary magnesium consumption. BMI was calculated as weight divided by the square of height (kg/m^2^). Participants were classified into three groups based on BMI: normal (<25 kg/m^2^), overweight (25 to <30 kg/m^2^), and obese (≥30 kg/m^2^). Supplementary Table 1 describes the detailed assessment criteria for smoking and drinking status. Diabetes was diagnosed based on fasting plasma glucose ≥7.0 mmol/L, or 2-h postprandial glucose ≥11.1 mmol/L, or glycated hemoglobin ≥6.5%, or self-reported history of diabetes, or self-reported use of hypoglycemic medications. Participants were considered to have CHD if they answered “yes” to the question, “Has a doctor or other health professional ever told you that you had coronary heart disease?”

### 2.5 Mortality

To assess the association of MDS with psoriasis mortality, the primary outcome was defined as all-cause mortality. Mortality data were recorded up to December 31, 2019 (https://www.cdc.gov/nchs/data-linkage/mortality.htm). The follow-up period was calculated from the date of the interview to December 31, 2019, or the date of death, whichever occurred first.

### 2.6 Statistical analysis

All analyses in this study included sample weights. First, differences in baseline characteristics between participants with and without psoriasis were compared. For continuous variables, t-tests or Wilcoxon rank-sum tests were used, while categorical variables were compared using chi-square tests. Continuous variables were described as weighted means and standard errors (SE), whereas categorical variables were expressed as frequencies and weighted percentages. Univariate logistic regression models were employed to explore the relationship between MDS and other potential risk factors with psoriasis. To account for confounding factors, multivariable logistic regression analysis was used to further assess the association between MDS and psoriasis prevalence. The results of the logistic regression were presented as odds ratios (OR) with 95% confidence intervals (CI). Four logistic regression models were constructed. Model 1 was unadjusted for any covariates. Model 2 was adjusted for sex, age, and race. Model 3 included adjustments for sex, age, race, education, PIR, and BMI. Model 4 further adjusted for smoking, drinking, diabetes, CHD, and dietary magnesium intake based on Model 3. Additionally, restricted cubic spline (RCS) was performed to explore potential nonlinear relationships between MDS and psoriasis prevalence after adjusting for all possible covariates included in Model 4. Subgroup analyses and interaction tests were conducted to examine potential heterogeneity across subgroups. In sensitivity analyses, multiple imputations were performed for missing covariates, and the analyses were repeated to verify the robustness of the results.

Additionally, Kaplan–Meier curves were used to compare survival differences among individuals with varying MDS levels. Cox regression analysis was employed to evaluate the association between MDS and all-cause mortality in psoriasis patients. The results of the Cox regression analysis were expressed as hazard ratios (HR) with 95% CI. The covariates adjusted in the model were consistent with those in the logistic regression model. Furthermore, subgroup analyses were conducted to further examine the robustness of the results.

All statistical analyses were performed using R software (version 4.3.2). A *p*-value < 0.05 was considered statistically significant.

## 3 Results

### 3.1 Baseline characteristics

This study ultimately included 17,883 participants, 506 of whom were diagnosed with psoriasis. Table 1 presents the baseline characteristics of the participants. The mean age of the participants was 44.8 years, among whom 50.0% were female. Participants with psoriasis and individuals without psoriasis demonstrated significant differences in several aspects, including age, race, BMI, smoking status, and MDS (*p* < 0.01). Participants with psoriasis were older and more likely to have ever smoked and be obese than individuals without psoriasis. The mean MDS for all participants was 0.77 ± 0.01. Notably, individuals with psoriasis had a significantly higher MDS compared to those without psoriasis (*p* = 0.001).

**Table 1:** Survey-weighted baseline characteristics of participants by psoriasis.

Characteristics	Total (*n* = 17,883)	Non-psoriasis (*n* = 17,377)	Psoriasis (*n* = 506)	*p* value
Age (years)	44.84 (0.29)	44.75 (0.29)	47.65 (0.76)	**<0.001**
Sex, n (%)				0.695
Female	8,839 (50.0)	8,580 (49.9)	259 (50.9)	
Male	9,044 (50.0)	8,797 (50.1)	247 (49.1)	
Race, *n* (%)				**<0.001**
Mexican American	2,667 (8.0)	2,627 (8.1)	40 (3.6)	
Non-Hispanic Black	3,849 (10.9)	3,781 (11.0)	68(6.1)	
Non-Hispanic White	8,402 (70.2)	8,085 (69.8)	317 (82.3)	
Other Hispanic	1,366(4.7)	1,327 (4.8)	39 (3.6)	
Other Race	1,599(6.2)	1,557 (6.3)	42 (4.4)	
Educational level, *n* (%)				0.081
Less than high school	1,448 (4.3)	1,418 (4.4)	30 (2.7)	
High school or equivalent	6,584 (33.2)	6,407 (33.3)	177 (30.2)	
College or above	9,851 (62.5)	9,552 (62.3)	299 (67.0)	
PIR	3.03 (0.04)	3.03 (0.04)	3.13 (0.08)	0.215
BMI (kg/m^2^)	28.80 (0.10)	28.76 (0.10)	30.21 (0.40)	**<0.001**
BMI group, *n* (%)				**0.002**
<25	5,380 (31.3)	5,270 (31.6)	110 (21.9)	
≥25 to<30	5,840 (32.8)	5,668 (32.7)	172 (36.0)	
≥30	6,663 (35.9)	6,439 (35.7)	224 (42.0)	
Smoking status, *n* (%)				**<0.001**
Never	9,743 (54.2)	9,525 (54.6)	218 (42.3)	
Former	4,056 (23.2)	3,893 (22.8)	163 (35.5)	
Now	4,084 (22.6)	3,959 (22.7)	125 (22.2)	
Drinking, *n* (%)				0.229
Never	2,245 (10.0)	2,191 (10.1)	54 (7.9)	
Former	3,026 (14.4)	2,924 (14.3)	102 (16.4)	
Now	12,612 (75.6)	12,262 (75.6)	350 (75.7)	
Diabetes, *n* (%)				0.261
No	15,107 (88.4)	14,700 (88.4)	407 (86.7)	
Yes	2,776 (11.6)	2,677 (11.6)	99 (13.3)	
Coronary heart disease, *n* (%)				0.082
No	17,302 (97.3)	16,830 (97.4)	472 (96.0)	
Yes	581(2.7)	547 (2.6)	34 (4.0)	
Dietary magnesium intake (mg)	307.64 (2.06)	307.61 (2.05)	308.55 (8.39)	0.909
MDS	0.77 (0.01)	0.77 (0.01)	0.96 (0.06)	**0.001**
MDS group, *n* (%)				**0.002**
0	8,607 (46.2)	8,413 (46.5)	194 (35.8)	
1	5,933 (36.1)	5,744 (35.9)	189 (42.2)	
2	2,330 (13.0)	2,245 (13.0)	85 (14.8)	
≥3	1,013 (4.7)	975 (4.7)	38 (7.2)	

Continuous variables are described using weighted means (standard errors). For categorical variables, unweighted *N* represents the study sample, while percentages reflect survey-weighted results.

PIR, poverty income ratio; BMI, body mass index; MDS, magnesium depletion score. Values presented in bold font denote statistical significance at the level of *p* < 0.05.

### 3.2 Univariate logistic regression analysis

Weighted univariate logistic regression analysis was performed to evaluate underlying risk factors for psoriasis. As shown in Supplementary Table 2, potential risk factors for psoriasis included older age, higher education, higher BMI, smoking, and higher MDS (*p* < 0.05). To remove the influence of confounding factors and further investigate the association between MDS and psoriasis, multivariable logistic regression models were adjusted for potential covariates, and dose–response relationships were explored by RCS.

### 3.3 Association between MDS and prevalence of psoriasis

**Figure 2:**
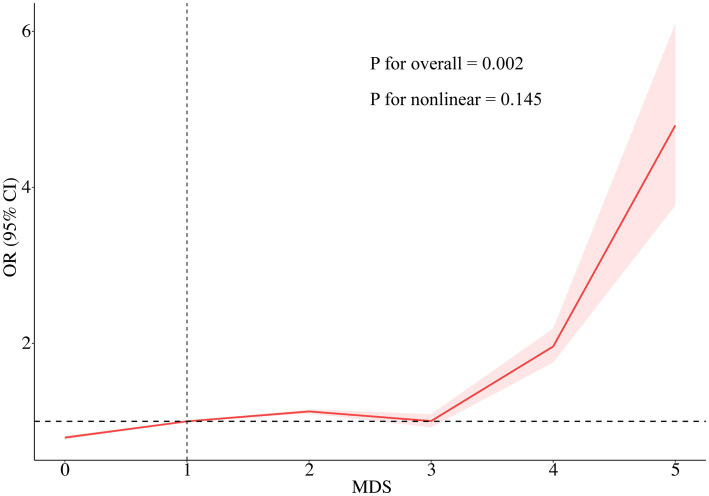
RCS of the association between MDS and psoriasis prevalence. The RCS model was adjusted for age, sex, race/ethnicity, education level, PIR, BMI, alcohol intake, smoking status, diabetes, coronary heart disease, and dietary magnesium intake. RCS, Restricted cubic spline; MDS, magnesium depletion score; OR, odds ratio; CI, confidence interval.

As illustrated in Table 2, weighted multivariate logistic regression analyses indicated that MDS was significantly and positively associated with the prevalence of psoriasis. When MDS was analyzed as a continuous variable in model 4, the risk of psoriasis increased by approximately 18% for each unit increase in MDS (OR = 1.18; 95% CI: 1.02–1.35; *p* < 0.05). When MDS was estimated as a categorical variable, in the crude model, participants with MDS ≥ 3 had an approximately 2.01-fold increased risk of psoriasis compared to those with a MDS of 0 (OR = 2.01; 95% CI: 1.27–3.18; *p* = 0.003). In Model 4, individuals with MDS ≥ 3 had a 1.75-fold higher risk of psoriasis than participants with an MDS of 0 (OR = 1.75; 95% CI: 1.05–2.92; *p* < 0.05). Furthermore, RCS analysis revealed an approximately linear positive correlation between MDS and psoriasis prevalence (*P* for nonlinear = 0.145) (Figure 2).

**Table 2:** Weighted multivariate logistic regression analyses between MDS and psoriasis.

Characteristics	Model 1		Model 2		Model 3		Model 4	
	OR (95% CI)	*P* value	OR (95% CI)	*P* value	OR (95% CI)	*P* value	OR (95% CI)	*P* value
MDS	1.25 (1.11, 1.41)	**<0.001**	1.23 (1.07, 1.42)	**0.004**	1.21 (1.06, 1.39)	**0.01**	1.18 (1.02, 1.35)	**0.02**
MDS group								
0	Reference	Reference	Reference	Reference	Reference	Reference	Reference	Reference
1	1.53 (1.17, 1.98)	**0.002**	1.43 (1.09, 1.88)	**0.01**	1.44 (1.09, 1.91)	**0.01**	1.42 (1.07, 1.88)	**0.01**
2	1.47 (1.02, 2.13)	**0.04**	1.39 (0.94, 2.06)	0.1	1.35 (0.91, 2.02)	0.13	1.33 (0.89, 1.98)	0.16
≥3	2.01 (1.27, 3.18)	**0.003**	1.98 (1.19, 3.27)	**0.01**	1.84 (1.12, 3.01)	**0.02**	1.75 (1.05, 2.92)	**0.03**
Trend test		**<0.001**		**0.006**		**0.012**		**0.021**

Model 1 was not adjusted for any covariates.

Model 2 was adjusted for age, sex, and race/ethnicity.

Model 3 was adjusted for age, sex, race/ethnicity, education level, PIR, and BMI.

Model 4 was adjusted for age, sex, race/ethnicity, education level, PIR, BMI, alcohol intake, smoking status, diabetes, coronary heart disease, and dietary magnesium intake.

OR, odds ratio; CI, confidence interval; MDS, magnesium depletion score; PIR, poverty income ratio; BMI, body mass index. Values presented in bold font denote statistical significance at the level of *p* < 0.05.

**Figure 3:**
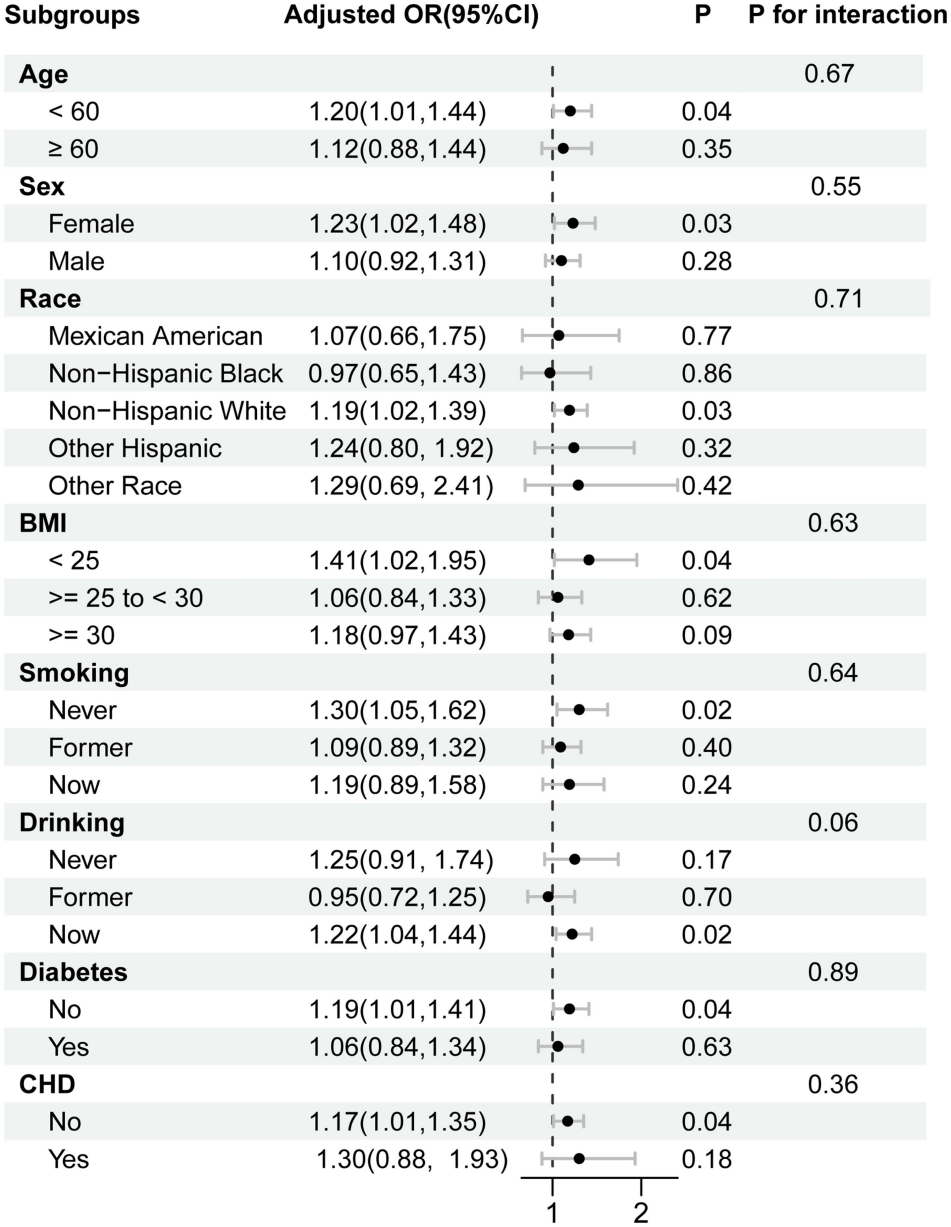
Subgroup analysis for the association between the MDS and psoriasis prevalence. Each stratification was adjusted for age, sex, race, educational levels, poverty income ratio, BMI, smoking, alcohol consumption, diabetes, coronary heart disease, and dietary magnesium intake, unless the variable was already used as a stratification factor. Abbreviations: OR, odds ratio; CI, confidence interval; MDS, magnesium depletion score; BMI, body mass index, CHD, coronary heart disease.

Subgroup analyses and interaction tests were conducted by dividing participants into eight subgroups based on age, sex, race, BMI, smoking, drinking, diabetes, and CHD. Figure 3 shows that MDS was significantly related to the prevalence of psoriasis in individuals under 60 years old, females, white, non-overweight or obese, never smokers, current drinkers, non-diabetics, and non-CHD patients (*p* < 0.05). However, we discovered no significant interaction between MDS and these possible confounders (all *P* for interaction > 0.05). The relationship between MDS and psoriasis prevalence was not significantly different across subgroups. The effect of magnesium deficiency on psoriasis is independent of these confounders. To further test the reliability and stability of these results, missing covariates were multiply interpolated for sensitivity analyses. This approach effectively reduced the risk of selection bias that might be triggered by the exclusion of participants with incomplete details. In the sensitivity analysis, MDS maintained a stable and significant positive association with the prevalence of psoriasis (Supplementary Table 3). The findings of subgroup analyses were also consistent with original analyses, as indicated in Supplementary Figure 1.

### 3.4 Association between MDS and all-cause mortality in patients with psoriasis

To assess the potential association between MDS and psoriasis mortality, we excluded participants who lacked follow-up information or did not suffer from psoriasis. The cohort study involved 505 patients with psoriasis. Baseline characteristics of the study population grouped by MDS level are shown in Supplementary Table 4. A total of 64 patients with psoriasis died from all causes during a median follow-up period of 104 months. Participants in the cohort with higher MDS tended to be older and more likely to have comorbid diabetes and CHD (*p* < 0.05). The Kaplan–Meier curves revealed notable variations in mortality across patients with varying degrees of MDS (*p* < 0.001) (Supplementary Figure 2). Notably, individuals with higher MDS had a significantly higher risk of mortality. As shown in Table 3, four Cox regression analysis models found a positive relationship between MDS and all-cause mortality in psoriasis patients. Even in Model 4 adjusted for all possible covariates, patients with MDS ≥ 3 faced a higher risk of all-cause mortality than individuals with MDS = 0 (HR = 3.87; 95% CI: 1.34–11.14; *p* = 0.01). Participants were categorized into five subgroups according to age, sex, diabetes, CHD, and dietary magnesium intake. Subgroup analyses and interaction tests were performed to explore possible heterogeneity. Supplementary Figure 3 shows the results. We found that the positive association between MDS and all-cause mortality remained stable across subgroups (all *P* for interaction > 0.05).

**Table 3:** Association between MDS and all-cause mortality in patients with psoriasis.

Characteristics	Model 1		Model 2		Model 3		Model 4	
	HR (95% CI)	*P* value	HR (95% CI)	*P* value	HR (95% CI)	*P* value	HR (95% CI)	*P* value
MDS	2.05 (1.69,2.49)	**<0.001**	1.52 (1.21,1.91)	**<0.001**	1.39 (1.09,1.78)	**0.01**	1.39 (1.04, 1.87)	**0.03**
MDS group								
0	Reference	Reference	Reference	Reference	Reference	Reference	Reference	Reference
1	1.38 (0.57,3.38)	0.48	0.99 (0.36,2.77)	0.99	1.01 (0.36,2.78)	0.99	1.00 (0.37,2.71)	0.99
2	4.87 (1.87,12.68)	**0.001**	2.89 (0.95,8.80)	0.06	2.59 (0.86,7.78)	0.09	2.74 (0.90,8.38)	0.08
≥3	10.71 (4.70,24.40)	**<0.001**	3.82 (1.51,9.70)	**0.005**	3.32 (1.25,8.80)	**0.02**	3.87(1.34,11.14)	**0.01**
Trend test		**<0.001**		**<0.001**		**0.002**		**0.003**

Model 1 was not adjusted for any covariates.

Model 2 was adjusted for age, sex, and race/ethnicity.

Model 3 was adjusted for age, sex, race/ethnicity, education level, PIR, and BMI.

Model 4 was adjusted for age, sex, race/ethnicity, education level, PIR, BMI, alcohol intake, smoking status, diabetes, coronary heart disease, and dietary magnesium intake.

HR, hazard ratio; CI, confidence interval; MDS, magnesium depletion score; PIR, poverty income ratio; BMI, body mass index. Values presented in bold font denote statistical significance at the level of *p* < 0.05.

## 4 Discussion

This study examined the relationship of MDS and the prevalence and all-cause mortality of psoriasis based on data from five NHANES cycles. The findings showed that MDS maintained significant and stable positive associations with psoriasis prevalence and all-cause mortality. These associations remained consistent across subgroups. The results provide new evidence supporting the environmental etiology of psoriasis from the perspective of systemic magnesium homeostatic imbalance, suggesting that systemic magnesium depletion may be directly involved in the pathological process of psoriasis. Our findings, if validated in future studies, could support the development of personalized dietary recommendations for psoriasis prevention. Similarly, the Dietary Inflammatory Index (DII), which quantifies the inflammatory potential of diet, has been linked to psoriasis incidence (19). Unlike the DII, which aggregates multiple dietary components, the MDS focuses specifically on magnesium status. It provides a more targeted assessment of magnesium depletion, potentially offering advantages in scenarios where magnesium deficiency is a hypothesized mechanistic driver of inflammation. Furthermore, serum vitamin D levels have been extensively studied in dermatological conditions, with deficiencies correlated with increased psoriasis severity (20). While serum vitamin D is a valuable biomarker, it represents a single snapshot. In contrast, the MDS combines both biochemical and dietary data, thereby providing a more comprehensive picture of nutrient status.

Magnesium is necessary for life and health (21). As a key cofactor of adenosine triphosphate, it plays an indispensable role in cellular energy metabolism. Furthermore, magnesium influences the metabolism of proteins, lipids, and carbohydrates in addition to serving as a cofactor for a variety of enzyme activities. Magnesium deficiency is a common nutritional deficiency state. Magnesium deficiency is influenced by various factors, including inadequate dietary intake, gastrointestinal absorption disorders, increased renal excretion, as well as medication use and chronic diseases. Due to unhealthy dietary patterns, many individuals often fail to meet the daily recommended intake of magnesium, leading to magnesium deficiency. The 2015–2020 Dietary Guidelines for Americans recommend an optimal dietary magnesium requirement of 320 mg per day for females and 420 mg per day for males under physiologic conditions. Importantly, the demand for magnesium may be higher under pathological conditions. Unfortunately, many Americans do not consume sufficient magnesium in their daily diets (22).

Although the precise mechanisms linking magnesium depletion to psoriasis pathogenesis remain to be fully elucidated, several plausible biological pathways, informed by the known role of magnesium in cellular functions, may explain our observed association. First, magnesium deficiency has been linked to elevated levels of substance P and neurogenic inflammation, which are known contributors to psoriasis flare-ups and pruritus (23). Moreover, magnesium deficiency is associated with inflammatory responses mediated by calcium, N-methyl-D-aspartate, and tumor necrosis factor-alpha, as well as increases in C-reactive protein (CRP) (24, 25). Besides, low magnesium levels trigger the activation of nuclear factor kappa B (NF-κB), a primary driver of the chronic inflammation characteristic of psoriasis (26). Magnesium modulates inflammatory signaling through multiple pathways and inhibits the generation of inflammatory mediators, thereby attenuating psoriasis. A meta-analysis that included 17 randomized controlled trials found that magnesium supplementation significantly reduced several inflammatory markers, particularly CRP (27). These findings indicate that the protective impact of magnesium supplementation in psoriasis is partially attributable to inflammation suppression effects. Interestingly, a recent study identified that a high-magnesium diet enhanced the number of regulatory T cells (Treg) in an IL-10-dependent manner through the mediation of the gut microbiota (28). Treg cells exert a protective role in psoriasis by suppressing inflammatory responses and maintaining immune homeostasis (29). Furthermore, magnesium ions specifically suppress the antigen-presenting function of human epidermal Langerhans cells *in vivo* and *in vitro*, contributing to the enhancement of psoriasis efficacy (30).

Second, magnesium has been demonstrated to inhibit oxidative stress and reduce impairment due to free radicals (31). Persistent magnesium deficiency exacerbates oxidative stress. Low magnesium levels trigger oxidative DNA modifications and impaired DNA repair, worsen skin inflammation, and stimulate hyperproliferation of keratinocytes (32). Psoriasis patients show abnormally elevated concentrations of reactive oxygen species (ROS) in areas of skin lesions (33). Mitochondria are the main site of intracellular ROS generation. When intracellular magnesium is deficient, magnesium translocation to mitochondria is suppressed, which in turn results in lower magnesium levels in mitochondria. Mitochondrial magnesium deficiency impairs the activity of the electron transport chain and interferes with the coupling efficiency of the respiratory chain, ultimately causing increased ROS generation (10). Interestingly, dietary magnesium supplementation significantly increased erythrocyte superoxide dismutase levels (34).

Third, magnesium has a significant effect in lipid metabolism, which is closely related to the pathomechanism of psoriasis by regulating sebum metabolism. Magnesium participates in fatty acid synthesis and cholesterol metabolism as a cofactor for several enzymes. Importantly, magnesium regulates phospholipase A2 (PLA2) activity. PLA2 is a vital enzyme involved in inflammatory responses and cellular signaling. Additionally, PLA2 is responsible for chronic epidermal hyperplasia and hyperkeratosis (35). Specifically, magnesium ions indirectly inhibit PLA2 activity by modulating intracellular calcium ion concentrations, reducing the release of arachidonic acid and its downstream production of proinflammatory lipid mediators, such as prostaglandin E2 and leukotriene B4 (36). It was found that magnesium supplementation contributed to lowering blood lipid levels (37). Magnesium ions reduce skin damage from external stimuli by regulating epidermal lipid composition and enhancing skin barrier function (38).

Magnesium condition was assessed by MDS in this study. MDS combines several major factors that influence magnesium reabsorption in the body, involving prescription medications, kidney function, and lifestyle habits. We discovered that greater MDS was associated with higher prevalence and all-cause mortality in psoriasis. Previous research has demonstrated that kidney failure acts as an independent risk factor for death (39). Notably, patients with psoriasis have a significantly elevated risk of chronic kidney disease (CKD) and end-stage renal disease compared to individuals without psoriasis (40, 41). Meanwhile, kidney diseases are likely to exacerbate psoriasis and significantly raise mortality risk from psoriasis (42). Hence, changes in eGFR-related markers in psoriasis patients need to be monitored to minimize renal damage. Additionally, people who use diuretics often have a combination of multiple underlying diseases, including cardiovascular disease, hypertension and diabetes, and other chronic disorders. Given this factor, people taking diuretics tend to face a higher risk of death. Moreover, excessive alcohol consumption hinders intestinal magnesium absorption, which is one of the key factors contributing to magnesium deficiency (43). Numerous evidences revealed that alcohol intake aggravates psoriasis progression (44). Besides, alcohol might also influence the absorption and metabolism of medications, reducing the efficacy of psoriasis and making it difficult to control (45). Therefore, it is essential to emphasize the negative effects of alcohol consumption on psoriasis to patients in clinical health education.

One effective way to enhance the management of psoriasis is to increase intake of protective key nutrients through dietary changes. The association between MDS and psoriasis may provide new perspectives on the prevention and treatment of psoriasis. First, psoriasis risk may be reduced by modifying diet to increase consumption of foods rich in magnesium. Green leafy vegetables, healthy grains, legumes, and nuts are excellent sources of magnesium. The Mediterranean diet focuses on legumes, fish, whole grains, olive oil, vegetables, fruits, and nuts high in B vitamins and magnesium. It has been demonstrated that following Mediterranean diet contributes to alleviating psoriasis (46). Second, magnesium supplementation may represent an effective tool to manage psoriasis, especially in high-risk populations such as patients taking PPIs and diuretics, and CKD patients. Serum vitamin D levels were found to be significantly lower in psoriasis patients compared to healthy controls (47). The severity of psoriasis was negatively correlated with serum vitamin D levels (48). Magnesium supplementation may indirectly benefit psoriasis patients by improving serum vitamin D levels.

This study utilized data from the NHANES database. The sample selected was representative and large. Secondly, potential confounding factors were adjusted, ensuring the reliability of the study findings. Furthermore, sensitivity analyses confirmed the robustness of the results. Nevertheless, this study has several limitations that need to be addressed. First, the cross-sectional design restricts causal inference, making it unable to demonstrate a causal association between MDS and psoriasis. Second, while several covariates were considered in this study, it cannot be guaranteed that all potential confounding variables have been completely excluded. Factors such as genetic predisposition, stress levels, or specific medications may influence outcomes. Third, the MDS is an indirect estimate of magnesium status. The lack of direct biomarker correlation means we cannot confirm a true cellular deficit or fully delineate the potential biological mechanisms linking magnesium status to psoriasis pathogenesis. Additionally, a key limitation of this study is its reliance on the U. S.-based NHANES database. Therefore, our findings may have limited generalizability to populations in other regions, particularly non-Western countries. Differences in genetic backgrounds, dietary patterns, environmental exposures, and healthcare systems could significantly influence both magnesium status and psoriasis, limiting the direct applicability of our results to these populations. To validate the universality of the results, large-scale prospective cohort studies in other populations are still needed. Finally, psoriasis was diagnosed based on self-reported data, which is susceptible to recall bias and misclassification. Although self-report is a practical for large surveys like NHANES, the absence of validation against clinical examinations or review of medical remains a limitation. Overall, although the current study lacks a clear biological mechanism to explain the interaction between MDS and psoriasis, these findings may provide new perspectives for the prevention and treatment of psoriasis.

## 5 Conclusion

This study revealed a positive association between MDS and the prevalence and all-cause mortality of psoriasis, suggesting that magnesium deficiency may be a risk factor for psoriasis and influence its prognosis. Therefore, early monitoring and management of MDS might benefit the prevention and treatment of psoriasis. Increasing dietary magnesium intake may serve as a simple and potentially beneficial adjunctive approach for managing psoriasis. Future studies should further examine the specific mechanisms by which magnesium homeostasis affects the physiopathological processes of psoriasis, particularly its role in T cell activation, cytokine release, and skin barrier function. Combined analysis of multi-omics data likely contributes to a more comprehensive understanding of the complex relationship between MDS and psoriasis. Furthermore, the observed results need to be further validated by prospective cohort studies and randomized controlled trials to facilitate their translation into clinical practice.

## Data Availability

Publicly available datasets were analyzed in this study. This data can be found at: https://www.cdc.gov/nchs/nhanes/.
