# Supplementary material for: Association between magnesium depletion score and prevalence and all-cause mortality of psoriasis among the US population
Source: Front Nutr. 2025 Sep 11;12:1598688. doi: 10.3389/fnut.2025.1598688 (PMC12460096; doi:10.3389/fnut.2025.1598688)
Supplement: Supplementary file 1 [file Supplementary_file_1.docx]

**Supplementary Materials**

**Supplementary Table 1** Definitions of smoking and drinking status.

| Personal behavioral variables | Definition |
| --- | --- |
| Smoking status | Never smokers: Individuals who have smoked fewer than 100 cigarettes throughout their lifetime.​  Former smokers: Those who have smoked more than 100 cigarettes in their lifetime but do not smoke at all now.​  Current smokers: Individuals who have smoked more than 100 cigarettes in their lifetime and either smoke on some days or smoke daily. |
| Alcohol consumption | Never drinkers: consumed < 12 drinks throughout their entire lifetime;  Former drinkers: consumed ≥ 12 drinks within a single year and did not drink last year, or did not drink last year but drank ≥ 12 drinks in lifetime;  Current drinkers: consumed ≥ 12 drinks in lifetime and had drunk alcohol in the last year. |

**Supplementary Table 2** Univariate weighted logistic regression of potential risk factors for psoriasis.

| character | OR (95% CI) | *P* value |
| --- | --- | --- |
| Age | 1.01(1.01,1.02) | **<0.001** |
| Sex |  |  |
| Female | Reference | Reference |
| Male | 0.96(0.80,1.17) | 0.7 |
| Race |  |  |
| Mexican American | Reference | Reference |
| Non-Hispanic Black | 1.26(0.78,2.03) | 0.35 |
| Non-Hispanic White | 2.67(1.76,4.05) | **<0.001** |
| Other Hispanic | 1.69(1.00,2.83) | 0.05 |
| Other Race | 1.60(0.95,2.69) | 0.08 |
| Education |  |  |
| Less than high school | Reference | Reference |
| High school or equivalent | 1.45(0.84,2.50) | 0.18 |
| College or above | 1.72(1.05,2.81) | **0.03** |
| PIR | 1.04(0.98,1.10) | 0.22 |
| BMI | 1.03(1.01,1.04) | **<0.001** |
| Smoking status |  |  |
| Never | Reference | Reference |
| Former | 2.02(1.59,2.55) | **<0.001** |
| Now | 1.26(0.96,1.67) | 0.1 |
| Drinking |  |  |
| Never | Reference | Reference |
| Former | 1.46(0.94,2.27) | 0.09 |
| Now | 1.28(0.92,1.79) | 0.15 |
| Diabetes |  |  |
| No | Reference | Reference |
| Yes | 1.17(0.88,1.56) | 0.26 |
| Coronary heart disease |  |  |
| No | Reference | Reference |
| Yes | 1.57(0.94,2.61) | 0.08 |
| Dietary magnesium intake | 1.00(1.00,1.00) | 0.91 |
| MDS | 1.25(1.11,1.41) | **<0.001** |
| MDS group |  |  |
| 0 | Reference | Reference |
| 1 | 1.53(1.17,1.98) | **0.002** |
| 2 | 1.47(1.02,2.13) | **0.04** |
| ≥3 | 2.01(1.27,3.18) | **0.003** |

Abbreviations: OR, odds ratio; CI, confidence interval; MDS, magnesium depletion score; PIR, poverty income ratio; BMI, body mass index.

**Supplementary Table 3** Weighted multivariable logistic regression analyses of MDS and psoriasis after multiple interpolation of covariates.

| Characteristics | Model 1 | | Model 2 | | Model 3 | | Model 4 | |
| --- | --- | --- | --- | --- | --- | --- | --- | --- |
|  | OR  (95% CI) | *P*  value | OR  (95% CI) | *P*  value | OR  (95% CI) | *P*  value | OR  (95% CI) | *P*  value |
| MDS | 1.27(1.13,1.42) | **<0.001** | 1.25(1.09,1.43) | **0.002** | 1.23(1.07,1.40) | **0.004** | 1.19(1.04,1.36) | **0.01** |
| MDS group |  |  |  |  |  |  |  |  |
| 0 | Reference | Reference | Reference | Reference | Reference | Reference | Reference | Reference |
| 1 | 1.49(1.15,1.93) | **0.003** | 1.39(1.06,1.83) | **0.02** | 1.39(1.05,1.85) | **0.02** | 1.36(1.02,1.81) | **0.03** |
| 2 | 1.52(1.07,2.15) | **0.02** | 1.42(0.97,2.07) | 0.07 | 1.38(0.93,2.04) | 0.1 | 1.31(0.88,1.93) | 0.18 |
| ≥3 | 2.13(1.39,3.25) | **<0.001** | 2.06(1.27,3.35) | **0.004** | 1.93(1.20,3.11) | **0.01** | 1.74(1.08,2.81) | **0.02** |
| Trend test |  | **<0.001** |  | **0.004** |  | **0.007** |  | **0.022** |

Model 1 was not adjusted for any covariates.

Model 2 was adjusted for age, sex, and race/ethnicity.

Model 3 was adjusted for age, sex, race/ethnicity, education level, PIR, and BMI.

Model 4 was adjusted for age, sex, race/ethnicity, education level, PIR, BMI, alcohol intake, smoking status, diabetes, coronary heart disease, and dietary magnesium intake.

Abbreviations: OR, odds ratio; CI, confidence interval; MDS, magnesium depletion score; PIR, poverty income ratio; BMI, body mass index.

**Supplementary Table 4** Weighted baseline characteristics of patients with psoriasis stratified by MDS level.

| Characteristics | total | 0 | 1 | 2 | ≥3 | P value |
| --- | --- | --- | --- | --- | --- | --- |
| Age (years) | 47.68(0.76) | 40.76(1.04) | 47.27(1.10) | 56.67(1.52) | 65.98(2.22) | **<0.001** |
| Sex, n (%) |  |  |  |  |  | 0.31 |
| Female | 259(51.0) | 98(51.2) | 99(49.0) | 40(48.0) | 22(67.5) |  |
| Male | 246(49.0) | 96(48.8) | 89(51.0) | 45(52.0) | 16(32.5) |  |
| Race, n (%) |  |  |  |  |  | 0.09 |
| Mexican American | 40( 3.6) | 19(5.6) | 14(2.8) | 7(2.5) | 0(0.0) |  |
| Non-Hispanic Black | 68( 6.1) | 34(9.1) | 21(4.7) | 10(4.4) | 3(3.6) |  |
| Non-Hispanic White | 317(82.5) | 103(74.8) | 127(85.9) | 57(86.0) | 30(93.4) |  |
| Other Hispanic | 39( 3.6) | 16(4.7) | 14(3.1) | 6(3.0) | 3(1.6) |  |
| Other Race | 41( 4.2) | 22(5.7) | 12(3.4) | 5(4.1) | 2(1.4) |  |
| PIR | 3.13(0.08) | 3.01(0.13) | 3.21(0.14) | 3.22(0.24) | 3.00(0.29) | 0.55 |
| BMI (kg/m^2^) | 30.22(0.40) | 29.67(0.77) | 29.52(0.48) | 32.40(1.12) | 32.60(2.23) | 0.06 |
| BMI group, n (%) |  |  |  |  |  | 0.28 |
| < 25 | 109(21.7) | 50(26.0) | 38(20.9) | 10(10.0) | 11(29.0) |  |
| >= 25 to < 30 | 172(36.1) | 57(32.3) | 73(39.8) | 33(39.4) | 9(26.6) |  |
| >= 30 | 224(42.2) | 87(41.7) | 77(39.2) | 42(50.5) | 18(44.4) |  |
| Education, n (%) |  |  |  |  |  | 0.82 |
| Less than high school | 30( 2.8) | 14(3.1) | 7(2.2) | 8(4.5) | 1(0.7) |  |
| High school or equivalent | 177(30.3) | 64(30.4) | 67(28.8) | 32(32.5) | 14(33.7) |  |
| College or above | 298(67.0) | 116(66.5) | 114(69.0) | 45(63.1) | 23(65.6) |  |
| Smoking status, n (%) |  |  |  |  |  | 0.1 |
| Never | 217(42.1) | 102(48.0) | 72(38.5) | 27(34.4) | 16(50.0) |  |
| Former | 163(35.6) | 43(27.6) | 60(37.1) | 40(46.5) | 20(44.9) |  |
| Now | 125(22.2) | 49(24.4) | 56(24.5) | 18(19.1) | 2( 5.1) |  |
| Drinking, n (%) |  |  |  |  |  | 0.72 |
| Never | 54( 7.9) | 25( 9.2) | 15( 5.9) | 9( 9.1) | 5(11.0) |  |
| Former | 102(16.5) | 39(16.2) | 41(18.6) | 17(14.5) | 5( 9.3) |  |
| Now | 349(75.6) | 130(74.6) | 132(75.6) | 59(76.3) | 28(79.7) |  |
| Diabetes, n (%) |  |  |  |  |  | **0.001** |
| No | 406(86.6) | 168(91.7) | 157(89.2) | 57(73.1) | 24(74.2) |  |
| Yes | 99(13.4) | 26( 8.3) | 31(10.8) | 28(26.9) | 14(25.8) |  |
| Coronary heart disease, n (%) |  |  |  |  |  | **<0.001** |
| No | 471(96.0) | 191(99.1) | 179(97.6) | 73(91.4) | 28(80.6) |  |
| Yes | 34( 4.0) | 3( 0.9) | 9( 2.4) | 12( 8.6) | 10(19.4) |  |
| Dietary magnesium intake (mg) | 308.22(8.41) | 303.48(14.06) | 313.87(15.47) | 318.71(17.85) | 277.53(23.10) | 0.45 |
| All-caused death, n (%) |  |  |  |  |  | **<0.001** |
| No | 441(90.6) | 184(95.8) | 171(94.0) | 65(81.1) | 21(64.5) |  |
| Yes | 64( 9.4) | 10( 4.2) | 17( 6.0) | 20(18.9) | 17(35.5) |  |

Continuous variables are described using weighted means (standard errors). For categorical variables, unweighted N represents the study sample, while percentages reflect survey-weighted results.

Abbreviations: PIR, poverty income ratio; BMI, body mass index; MDS, magnesium depletion score.

**Supplementary Figure 1** Subgroup analysis for the association between the MDS and psoriasis after multiple interpolation of covariates.


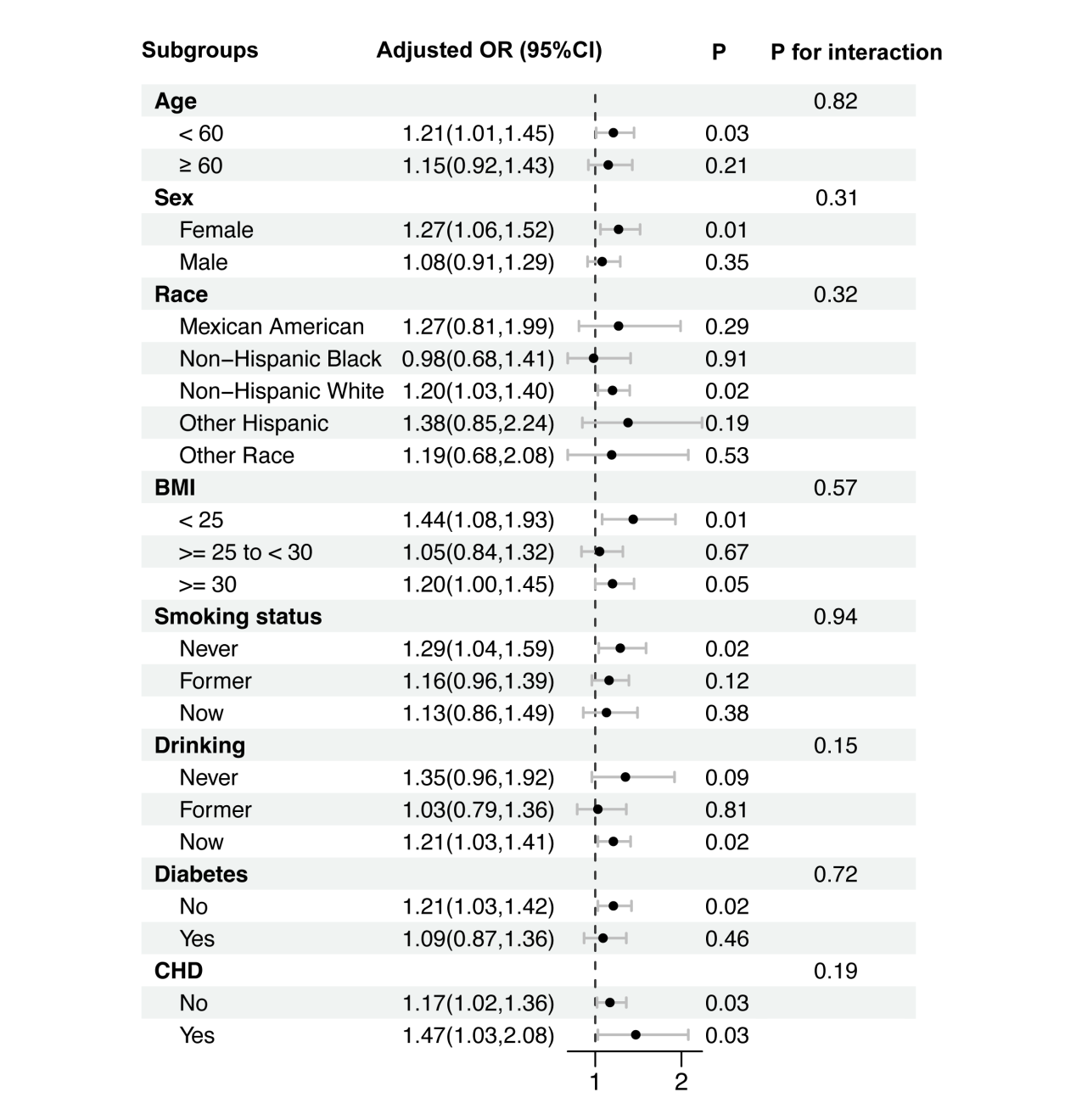


Each stratification was adjusted for age, sex, race, educational levels, poverty income ratio, smoking, alcohol consumption, diabetes, coronary heart disease, and dietary magnesium intake, unless the variable was already used as a stratification factor.

Abbreviations: OR, odds ratio; CI, confidence interval; MDS, magnesium depletion score; BMI, body mass index, CHD, coronary heart disease.

**Supplementary Figure 2** Kaplan–Meier survival estimates for all-cause mortality in patients with psoriasis.

**Supplementary Figure 3** Subgroup analysis of the relationship between MDS and all-cause mortality in psoriasis.

Each stratification was adjusted for age, sex, race, educational levels, poverty income ratio, smoking, alcohol consumption, diabetes, coronary heart disease, and dietary magnesium intake, unless the variable was already used as a stratification factor.

Abbreviations: HR, hazard ratio; CI, confidence interval; MDS, magnesium depletion score; CHD, coronary heart disease.
